# Network Pharmacology, Molecular Docking, and Molecular Dynamics Simulation to Elucidate the Molecular Targets and Potential Mechanism of *Phoenix dactylifera* (Ajwa Dates) against Candidiasis

**DOI:** 10.3390/pathogens12111369

**Published:** 2023-11-18

**Authors:** Mohd Adnan, Arif Jamal Siddiqui, Syed Amir Ashraf, Fevzi Bardakci, Mousa Alreshidi, Riadh Badraoui, Emira Noumi, Bektas Tepe, Manojkumar Sachidanandan, Mitesh Patel

**Affiliations:** 1 Department of Biology, College of Science, University of Ha’il, Ha’il 55473, Saudi Arabia; drmohdadnan@gmail.com (M.A.);; 2 Medical and Diagnostic Research Centre, University of Ha’il, Ha’il 55473, Saudi Arabia; 3 Department of Clinical Nutrition, College of Applied Medial Sciences, University of Ha’il, Ha’il 55473, Saudi Arabia; 4 Department of Molecular Biology and Genetics, Faculty of Science and Literature, Kilis 7 Aralik University, Kilis TR-79000, Turkey; 5 Department of Oral Radiology, College of Dentistry, University of Ha’il, Ha’il 55473, Saudi Arabia; 6 Department of Biotechnology, Parul Institute of Applied Sciences and Centre of Research for Development, Parul University, Vadodara 391760, India

**Keywords:** *Phoenix dactylifera*, Ajwa dates, candidiasis, fungal infection, network pharmacology, molecular dynamics

## Abstract

Candidiasis, caused by opportunistic fungal pathogens of the *Candida* genus, poses a significant threat to immunocompromised individuals. Natural compounds derived from medicinal plants have gained attention as potential sources of anti-fungal agents. Ajwa dates (*Phoenix dactylifera* L.) have been recognized for their diverse phytochemical composition and therapeutic potential. In this study, we employed a multi-faceted approach to explore the anti-candidiasis potential of Ajwa dates’ phytochemicals. Utilizing network pharmacology, we constructed an interaction network to elucidate the intricate relationships between Ajwa dates phytoconstituents and the *Candida*-associated molecular targets of humans. Our analysis revealed key nodes in the network (STAT3, IL-2, PTPRC, STAT1, CASP1, ALB, TP53, TLR4, TNF and PPARG), suggesting the potential modulation of several crucial processes (the regulation of the response to a cytokine stimulus, regulation of the inflammatory response, positive regulation of cytokine production, cellular response to external stimulus, etc.) and fungal pathways (Th17 cell differentiation, the Toll-like receptor signaling pathway, the C-type lectin receptor signaling pathway and necroptosis). To validate these findings, molecular docking studies were conducted, revealing the binding affinities of the phytochemicals towards selected *Candida* protein targets of humans (ALB–rutin (−9.7 kJ/mol), STAT1–rutin (−9.2 kJ/mol), STAT3–isoquercetin (−8.7 kJ/mol), IL2–β-carotene (−8.5 kJ/mol), CASP1–β-carotene (−8.2 kJ/mol), TP53–isoquercetin (−8.8 kJ/mol), PPARG–luteolin (−8.3 kJ/mol), TNF–βcarotene (−7.7 kJ/mol), TLR4–rutin (−7.4 kJ/mol) and PTPRC–rutin (−7.0 kJ/mol)). Furthermore, molecular dynamics simulations of rutin–ALB and rutin-STAT1 complex were performed to gain insights into the stability and dynamics of the identified ligand–target complexes over time. Overall, the results not only contribute to the understanding of the molecular interactions underlying the anti-fungal potential of specific phytochemicals of Ajwa dates in humans but also provide a rational basis for the development of novel therapeutic strategies against candidiasis in humans. This study underscores the significance of network pharmacology, molecular docking and dynamics simulations in accelerating the discovery of natural products as effective anti-fungal agents. However, further experimental validation of the identified compounds is warranted to translate these findings into practical therapeutic applications.

## 1. Introduction

Candidiasis, a group of fungal infections caused by species of the *Candida* genus, has emerged as a prominent healthcare concern with increasing incidence and clinical significance [1,2,3]. *Candida* species are commensal organisms found in various mucosal and cutaneous sites in the human body. However, under certain conditions, these fungi can transition from harmless commensals to opportunistic pathogens, leading to a range of clinical manifestations, from mild mucosal infections to life-threatening systemic diseases [4,5,6]. The *Candida* genus encompasses a diverse array of species, with *Candida albicans* being the most prevalent and well-studied one. Other species, such as *Candida glabrata*, *Candida tropicalis* and *Candida auris* have received attention due to their growing impact, particularly in immunocompromised and critically ill patients [7,8]. Candidiasis is associated with a wide spectrum of clinical presentations, including oral thrush, vaginal infections, cutaneous infections, and invasive bloodstream infections, the latter of which have alarmingly high mortality rates [9,10,11,12].

Several factors contribute to the rising challenge of candidiasis. Immunocompromised individuals, such as those with HIV/AIDS undergoing cancer treatments, or organ transplant recipients, are at heightened risk [13,14,15]. Additionally, the increasing use of invasive medical interventions, broad-spectrum antibiotics, and immunosuppressive therapies has created favorable conditions for *Candida* species to flourish and cause infections [16]. Moreover, the emergence of drug-resistant strains, particularly in hospital settings, further complicates treatment options [17,18]. The treatment of candidiasis relies primarily on anti-fungal agents, including azoles, echinocandins, and polyenes [19]. However, the efficacy of these drugs is becoming compromised due to the development of anti-fungal resistance, which emphasizes the need for innovative treatment strategies. Furthermore, the limited therapeutic options for certain species, such as the multidrug-resistant *Candida auris*, highlight the urgency for novel interventions [17,18,20].

Natural products have garnered increasing attention for their potential as sources of novel antimicrobial agents [21,22,23]. Among these is the Ajwa date (*Phoenix dactylifera* L.), a popular fruit known for its nutritional and medicinal value [24,25]. Ajwa dates have been traditionally used for their health benefits and have exhibited various bioactivities, including antimicrobial effects [26,27]. Previous studies have reported their inhibitory effects against various microorganisms, including fungi [28,29,30]. However, the intricate molecular mechanisms underlying their antimicrobial activity, particularly against *Candida* species in humans, remain largely unexplored. Network pharmacology, a systems biology-based approach, offers a holistic perspective on the interactions between bioactive compounds and their targets within complex biological systems [31]. This approach allows for the identification of potential therapeutic targets, elucidation of molecular pathways, and prediction of synergistic interactions between bioactive compounds [32,33]. This study aims to employ an integrative network pharmacology approach to elucidate the multi-target pharmacological mechanism underlying the antimicrobial activity of Ajwa dates against candidiasis in humans. Through the integration of bioinformatics, computational analyses, and experimental validation, we aim to identify key bioactive compounds in Ajwa dates, predict potential target proteins within humans for the *Candida* species and elucidate the molecular pathways through which these compounds can exert their antimicrobial effects.

## 2. Materials and Methods

### 2.1. Identifying the Potential Targets of Compounds and Diseases

In the present study, phytochemical constituents of Ajwa dates known for their antimicrobial activity were selected from the literature [34,35,36]. The PubChem database (http://pubchem.ncbi.nlm.nih.gov/ (accessed on 15 February 2023)) was used as the source of information about these molecules structure, molecular weights, and canonical smiles, along with the corresponding .sdf files (accessed on 16 February 2023). For the purpose of retrieving targets for phytochemical constituents of Ajwa dates related to the species *Homo sapiens*, different databases including SwissTargetPrediction and PharmMapper were utilized. For the purpose of standardizing the names of the target proteins, the UniProtKB database (https://www.uniprot.gov/ (accessed on 16 February 2023)) was consulted [37,38]. The targets related to candidiasis in humans were identified by searching for keywords such as “candidiasis” and “candida infection” in the GeneCards database (http://www.genecards.org/ (accessed on 16 February 2023)), the Online Mendelian Inheritance in Man database (OMIM, https://omim.org/ (accessed on 16 February 2023)) and the gene–disease associations database (DisGeNET, http://www.disgenet.org/) (accessed on 16 February 2023) [39,40]. Using the UniProt database (https://www.UniProt.org/ (accessed on 16 February 2023)), the target protein names were converted into gene names [41]. Following the removal of repetitive targets, all candidiasis targets of humans were acquired.

### 2.2. Finding and Acquiring Potential Targets

Several potential targets were identified in the study, including those that are predicted for phytochemical constituents of Ajwa dates as well as those connected with candidiasis in humans. FunRich tool version 3.1.3 was used to construct Venn diagrams for analyzing common targets [42]; the Swiss target prediction database was used for information regarding the classes of potential protein targets [http://www.swisstargetprediction.ch/error_page.php?error=1/ search (accessed on 15 February 2023)].

### 2.3. Construction and Analysis of Protein–Protein Interaction Network 

The STRING database [https://string-db.org/ (accessed on 20 February 2023)] was utilized to study protein–protein interactions (PPIs) of potential targets [43]. According to the parameter settings, the analysis was performed at a confidence level of 0.400, while a false discovery rate (FDR) stringency of 5% was assigned. Cytoscape software (Version 3.9.1) was used to build and analyze a PPI network of the potential targets [44]. The nodes of the network were analyzed for their topological features and a range of possible targets were selected by taking into account three parameters, namely “degree”, “betweenness centrality” and “closeness centrality”. These three parameters were used to estimate the topological properties of the nodes.

### 2.4. Findings of Hub-Genes and GO-KEGG Pathway Enrichment Analysis

Using the cytohubba plugin of Cytoscape tool, the top ten hub genes in the network were identified, and based on the maximal clique centrality (MCC) topological analysis, the top ten hub genes in the network were predicted. In order to analyze the biological functions of target proteins and pathways associated with diseases, a functional enrichment analysis was performed on the DAVID database [https://david.ncifcrf.gov/ (accessed on 25 February 2023)] [45]. In order to visualize the enriched GO terms and pathways, a false discovery rate (FDR) of less than 0.05 was used. In order to summarize the top ten most insightful GO terms (BP, CC and MF) using bioinformatics tools, a bubble graph was generated using SRplot [https://www.bioinformatics.com.cn/ (accessed on 25 February 2023) and a top ten KEGG pathway map was also generated using SRplot.

### 2.5. Molecular Docking Analysis

An investigation of the interaction between phytochemical constituents of Ajwa dates and identified candidiasis targets of humans was conducted using AutoDock Vina [46]. From the PubChem database, the 3D structures of phytochemical constituents were downloaded. The 3D structures of each compound were converted from .sdf into .pdb using Open Babel 3.1.1. Energy minimization was performed using Avogadro using an MMFF94 force field. In total, 5000 steps were taken to be optimized using the Steepest Descent algorithm. In the process of energy minimization, the structure was updated at every step and when the energy difference was less than 0.1, minimization was terminated, and then the .PDB file was saved. Protein 3D crystal structures were downloaded from RCSB-PDB database ALB (PDB ID: 1AO6), STAT3 (PDB ID: 1BG1), STAT1 (PDB ID: 1BF5), IL2 (PDB ID: 1M48), CASP1 (PDB ID: 3DCY), TP53 (PDB ID: 6PZP), TNF (PDB ID: 2AZ5), PTPRC (PDB ID: 5FMV), TLR4 (PDB ID: 2Z65) and PPARG (PDB ID: 6TSG). Water molecules were deleted from the crystal structure. The protein structure was then charged with a Kollman charge and hydrogen was added. The coordinates of the proteins were saved in a .pdb file. The Open Babel tool was then used to convert all structures from .pdb into .pdbqt. The molecular docking analysis was carried out via AutoDock 4.2.6 [46]. All the parameters used for the docking of phytochemical constituents of Ajwa dates with the target proteins of humans against candidiasis were kept the same, except for the grid center, which differed for each protein inside the grid box. Auto Grid was used for the preparation of the grid map, using a grid box. The grid size was set to X = 74.08, Y = 61.54 and Z = 80.45 for ALB; X = 37.42, Y= 38.38 and Z = 48.30 for IL2; X= 57.57, Y = 49.16 and Z = 57.01 for PPARG; X = 105.82, Y = 115.82 and Z = 75.76 for PTPRC; X = 123.35, Y = 87.09 and Z = 78.49 for STAT1; X = 100.26, Y = 62.06 and Z = 121.15 for STAT3; X = 38.89, Y = 40.61 and Z = 76.95 for TLR4; X = 42.98, Y = 40.70 and Z = 37.44 for TNF; X = 48.44, Y = 51.47 and Z = 54.09 for TP53; X = 54.30, Y = 50.27 and Z = 66.25 points for CASP1, for all proteins. Grid spacing was kept to 0.375 Å for all the proteins. The grid center for ALB was designated at dimensions (x, y, and z) of 29.5302, 31.8382, and 23.5064; that for IL2 was designated at (x, y, and z) 9.4214, 12.7182, and 6.5658; that for PPARG was designated at (x, y and z) −10.0619, 1.9499, and −26.9904; that for PTPRC was designated at (x, y, and z) −4.3593, 15.8529 and 18.7986; that for STAT1 was designated at (x, y, and z) 71.9873, 46.5916, and 79.496; that for STAT3 was designated at (x, y, and z) 117.3573, 87.4948, and 31.1853; that for TLR4 was designated at (x, y, and z) 22.6019, 5.1926, and 31.938; that for TNF was designated at (x, y, and z) −26.3998, 65.8979, and 41.9649; that for TP53 was designated at (x, y, and z) 22.9991, 37.3443; and 3.7189 and that for CASP1 was designated at (x, y, and z) −12.6912., −30.7536, and −9.2255. The grid box was created in such a way that it enclosed the entire binding site of each protein and provided enough space for the translation and rotation of ligands. The generated docked conformation was ranked according to predicted binding energy, and the topmost binding energy’s docked conformation was analyzed using PyMOL and Discovery Studio Visualizer [47]. By using Discovery Studio Visualizer, it was possible to explore the types of interactions, the participating residuals, and the atomic coordinates involved.

### 2.6. Molecular Dynamics Simulation 

A further study was undertaken to understand how ligands behave within the binding pocket of receptors as a result of their time-dependent conformational stability, which was based on the MD studies. The usefulness of this method has been demonstrated in several studies, including the identification of new inhibitors in a variety of applications [48,49,50,51]. As part of this study, MD analyses were conducted using GROMACS version 2019.4 [52]. In order to conduct MD studies, the GROMOS force field was used. From the ATB server, the topology of the chosen ligand was determined in order to obtain the coordinates of the force field. This system was optimized by minimizing the vacuum in 1500 steps by using a steepest descent algorithm. Following that, the complex structures were solvated in a cubic periodic box of 0.5 nanometers using a simple point charge water model (SPC). In order to maintain an appropriate salt concentration (0.015 M) in the complex systems, appropriate numbers of Na^+^ and Cl^-^ counterions were added. A leap frog algorithm was used to equilibrate NVT and NPT for 100 ps steps. Following equilibration, the production MD was applied to the complex of the solvated protein and ligand for 100 nanoseconds. After removing periodic boundary conditions from the MD run, a trajectory file was further analyzed. Data were analyzed using the Chimera package. XMGRACE was used to generate the diagrams (https://plasma-gate.weizmann.ac.il/Grace/ (accessed on 1 March 2023)).

### 2.7. Binding Free Energy Calculations

By using the Poisson-Boltzmann surface (PBSA) method of molecular mechanics (MM), binding free energy calculations were performed. The MM-PBSA method has been widely used in drug discovery to calculate the solvation-based score of protein-ligand interactions. Solvation free energy (polar and non-polar) and vacuum potential energy were used to determine the solvation-based binding free energy. Non-polar and polar solvation energy terms have been calculated using Poisson-Boltzmann equations and solvent accessible surface areas (SASA). In order to estimate the affinity between a ligand and a receptor, the Poisson–Boltzmann equation was used. By utilizing a van der Waals contact probe, the SASA method was used to predict which solvent was surrounding a protein surface. To calculate the MM-PBSA, a script in the G_MMPBSA module was used that uses the AMBERTOOLS [53] software in the backend.

The solvation-based free energy of binding (∆G_binding_) was calculated using the equations given below:

∆G_binding_ = ∆G_MM (Potential energy vaccum)_ + ∆G_sol (Solvation effects)_
Where, ∆G_MM_ = ∆G_Coloumb (electrostatic interaction)_ + ∆G_vdw_ and
∆Gsol = ∆G_polar_ + ∆G_nonpolar_

## 3. Results

### 3.1. Prediction and Screening of Compound-Diseases Targets

**Figure 1:**
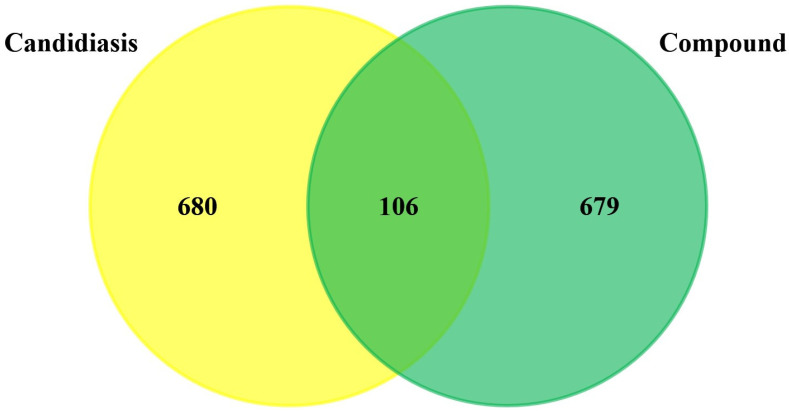
Venn diagram showing common targets between phytochemical constituents of Ajwa dates and candidiasis in humans.

According to the literature search, 17 phytochemical constituents of Ajwa dates were selected, and their detailed information was retrieved from the PubChem database in order to be analyzed through the use of SwissTargetPrediction and PharmMapper databases (Table 1). Once duplicate targets were removed from the predicted targets, a screening of 785 potential targets was carried out for further evaluation. A database of human genomes was used in order to gather the targets that are related to the development of candidiasis in humans. There were 128, 95, and 892 identified targets in total in OMIM, DisGeNET, and GeneCards, respectively. After the removal of duplicate entries from these databases, in total, 786 candidiasis targets in humans were identified. The intersection of these targets with component targets resulted in 106 intersection targets (Figure 1). 

### 3.2. Compound–Disease Common Target Network Construction and Analysis

**Figure 2:**
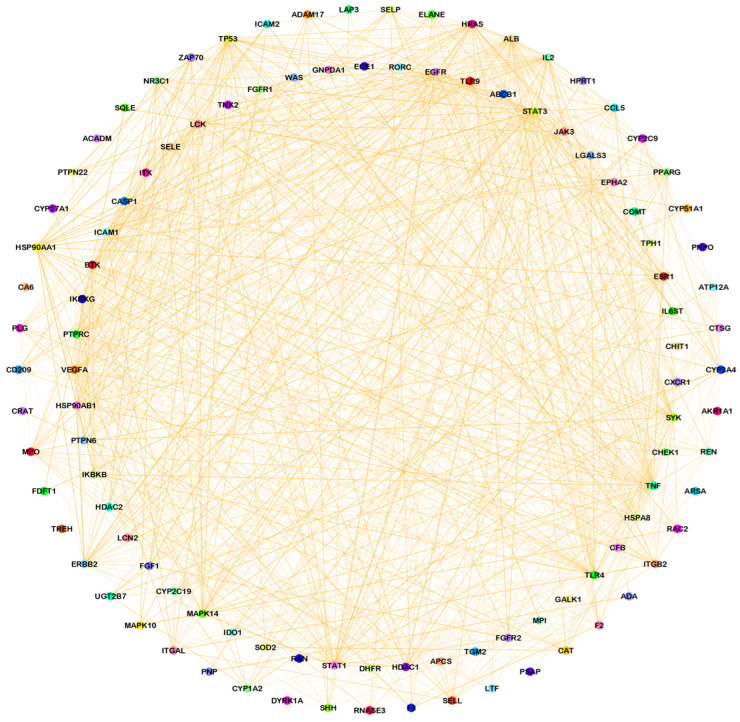
A common protein target network of phytochemical constituents of Ajwa dates and candidiasis-associated targets of humans constructed using Cytoscape (different-color circles represent common target proteins and orange-color edges represent interactions between common targets).

**Figure 3:**
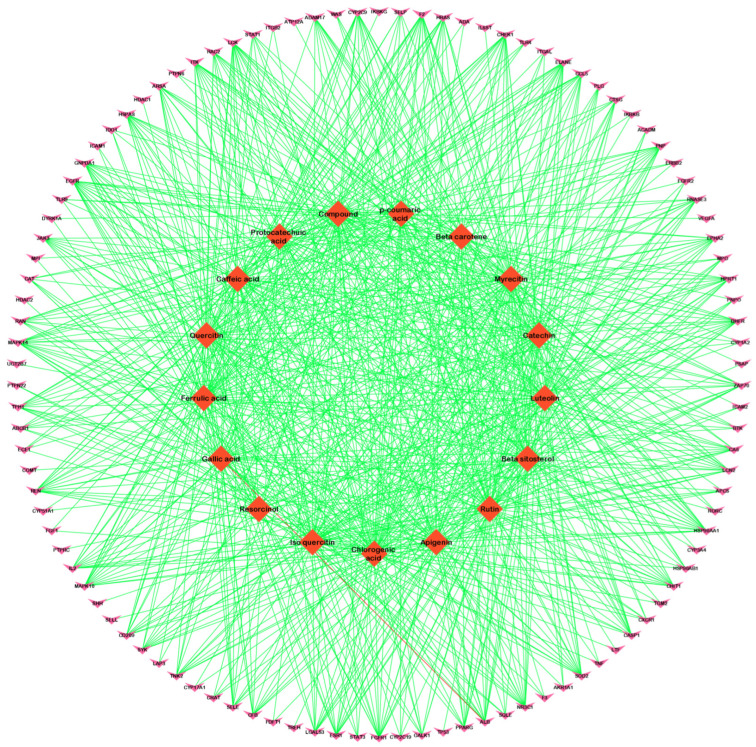
An Ajwa date–phytochemical constituent–intersected candidiasis protein target network, which is a network between the phytochemical constituents of Ajwa dates and the intersected genes (the pink ‘V’ shape represents common protein targets, orange diamonds represent phytochemical constituents of Ajwa dates and green-color edges denote the association between targets).

**Figure 4:**
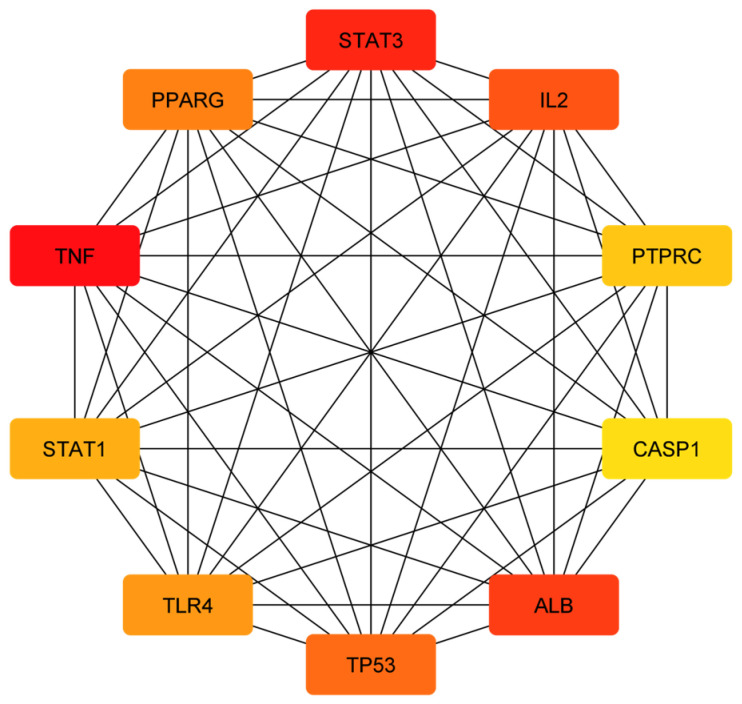
A PPI network of identified hub targets from the obtained common targets of phytochemical constituents of Ajwa dates and candidiasis for humans. A gradient of orange shades indicates the centrality degree of the nodes. Darker shades represent nodes with higher-degree centrality (i.e., they have more connections (edges) to other nodes in the network). Conversely, lighter or paler shades indicate nodes with lower-degree centrality (i.e., signifying fewer connections).

**Figure 5:**
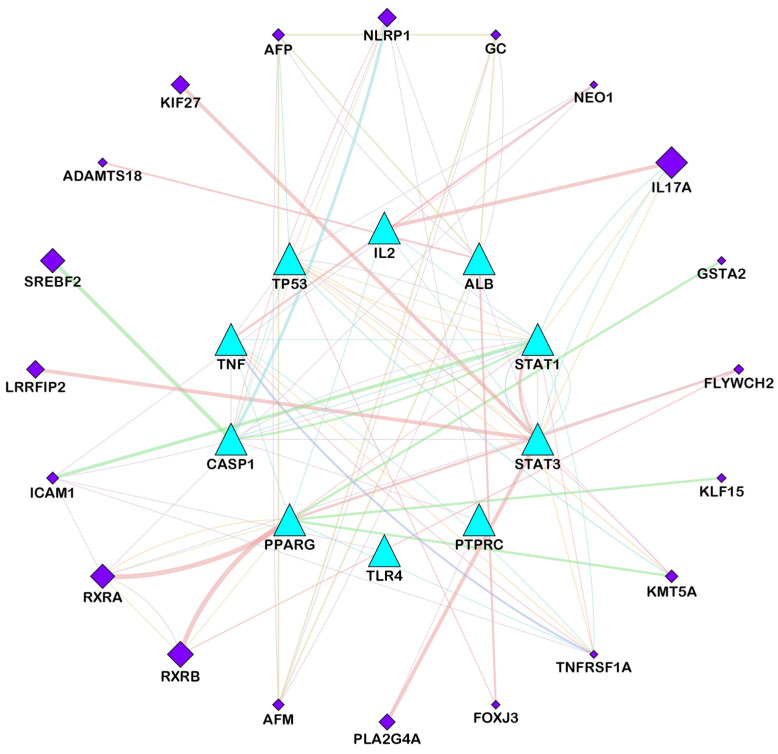
Network of hub targets against candidiasis in humans analyzed using GeneMANIA. The functional association of targets was analyzed, and connecting lines with different colors represent different correlations. Targets (cyan-color triangles) on the inner side were submitted as query terms in searches. Nodes (purple-color diamonds) on the outer side represent targets associated with query targets. (Peach-pink-color edges represent physical interactions, purple-color edges represent co-expression, green-color edges represent genetic interactions, cyan-color edges represent pathways, sand-brown-color edges represent colocalization, and orange-color edges represent shared protein domains).

The relationships between target genes were analyzed by using a PPI network. During the course of this process, potential targets were entered into the STRING database as the first step, and then after receiving the data, we used Cytoscape version 3.9.1 to analyze and visualize the data. A network of identified common targets was created with the help of Cytoscape version 3.9.1, which consisted of 106 nodes and 958 edges (Figure 2). Similarly, a network of a component intersection target was also created via Cytoscape version 3.9.1, which consisted of 106 nodes and 330 edges (Figure 3). The significance of each node within a complex network was estimated using three parameters: the degree, closeness and centrality between the nodes in the network (Table 2 and Table 3). These three parameters were used in order to estimate how significant each node was compared with how significant other nodes were in the network. Several genes that have been reported to play a crucial role in the development of candidiasis in humans were identified in this study. As a result of these findings, it appears that the anti-fungal activity of Ajwa dates exhibited by a variety of phytochemical constituents can be attributed to the activities of these key targets. According to the topology properties of the network, there were ten targets in the network, corresponding to STAT3, IL-2, PTPRC, STAT1, CASP1, ALB, TP53, TLR4, TNF and PPARG, the interaction of which with each other via edges are presented in Figure 4. The phytochemical constituents of Ajwa dates may be able to target these ten targets in order to effectively fight candidiasis in humans by targeting them. Additionally, we used the GeneMANIA tool to export the identified protein targets of humans into a PPI network so we could see what kind of relationships there might be between the identified target proteins and others in the network. According to the results, the percentage represents the weight that is given to the interactions in the network. Based on the analysis of all the interactions between targets in the network, it was estimated that 4.91% of the interactions involved co-expressions and 70.38% of them involved physical interactions between the targets. The results of the study also revealed that there was a correlation between genetic interactions (10.80%), predicted (1.14%) and colocalization (2.92%) (Figure 5).

### 3.3. Analysis of Functional and Pathway Enrichment

**Figure 6:**
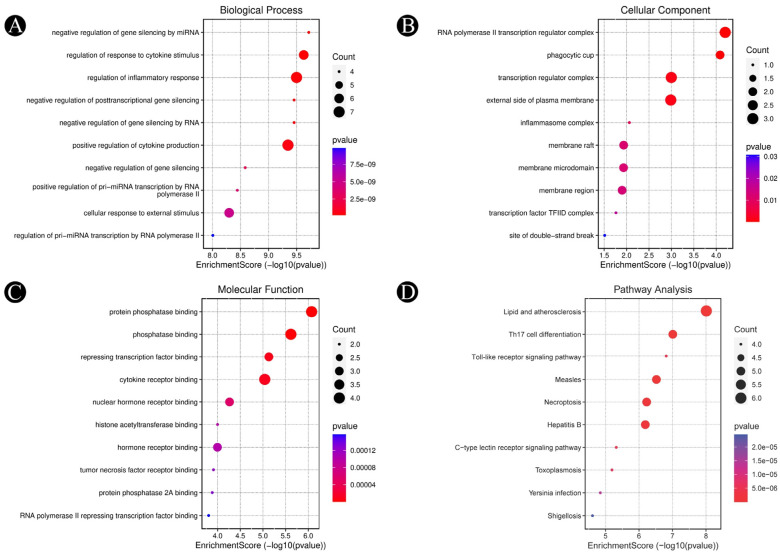
GO enrichment and KEGG pathway analyses of identified hub target proteins (*p*-value ≤ 0.05). (**A**) The top 10 biological processes, (**B**) the top 10 cellular components, (**C**) the top 10 molecular functions and (**D**) the top 10 KEGG pathways. The color scales indicate the different thresholds for the *p*-values, and the sizes of the dots represent the number of targets corresponding to each term.

GO and KEGG analyses were performed using the DAVID database to enrich the top 10 intersected targets. In the course of screening, 705 items in total were obtained pertaining to biological processes (BP), molecular functions (MF), and cellular components (CC) (Figure 6A–C), along with a *p*-value of < 0.05, as screening conditions. The total number of items obtained pertaining to biological process was 705, the number of items obtained pertaining to molecular function was 79, and the number of items obtained pertaining to cellular components was 25. There is a possibility that the phytochemical constituents in Ajwa dates might be involved in inhibiting candidiasis in humans by acting on the regulation of the response to a cytokine stimulus, regulation of the inflammatory response and positive regulation of cytokine production and the cellular response to an external stimulus via molecular functions such as protein phosphatase binding, cytokine receptor binding, the repression of transcription factor binding, nuclear hormone receptor binding, tumor necrosis factor receptor binding, histone acetyltransferase binding in cellular compartments such as transcription regulator complex, inflammasome complex, membrane raft, membrane microdomain, membrane region, and transcription factor TFIID complex. As a result of the KEGG pathway enrichment analysis, 123 enrichment results were obtained. Among them, Th17 cell differentiation, the Toll-like receptor signaling pathway, the C-type lectin receptor signaling pathway and necroptosis are closely associated with candidiasis in humans and are in accordance with the enrichment results of GO. The significance of KEGG pathways and gene pathways was demonstrated with *p*-values of < 0.05. A SRplot was used to analyze the first ten components (Figure 6D). Statistically, ten proteins were found to have a significant frequency of participation in each of the first 10 pathways, which served as an indication that these proteins played a key role in the enrichment pathway. The ten core proteins are STAT3, IL2, PTPRC, STAT1, CASP1, ALB, TP53, TLR4, TNF and PPARG.

### 3.4. Molecular Docking Analysis

**Figure 7:**
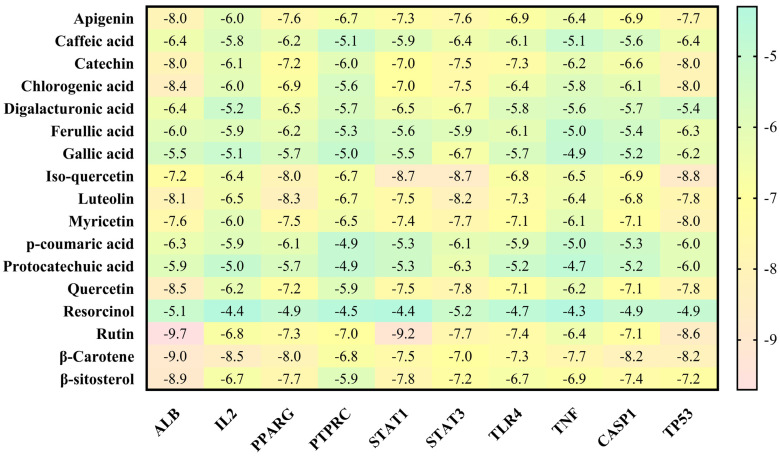
Binding affinities of the top-rated pose of the ligand–receptor complex.

**Figure 8:**
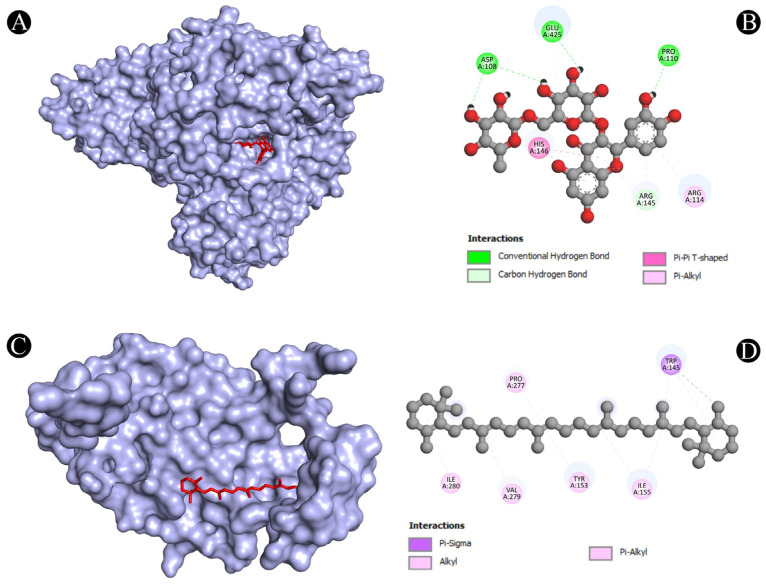
(**A**,**B**) Visualization of docking analysis of ALB and rutin, and (**C**,**D**) visualization of docking analysis of CASP1 and β-carotene.

**Figure 9:**
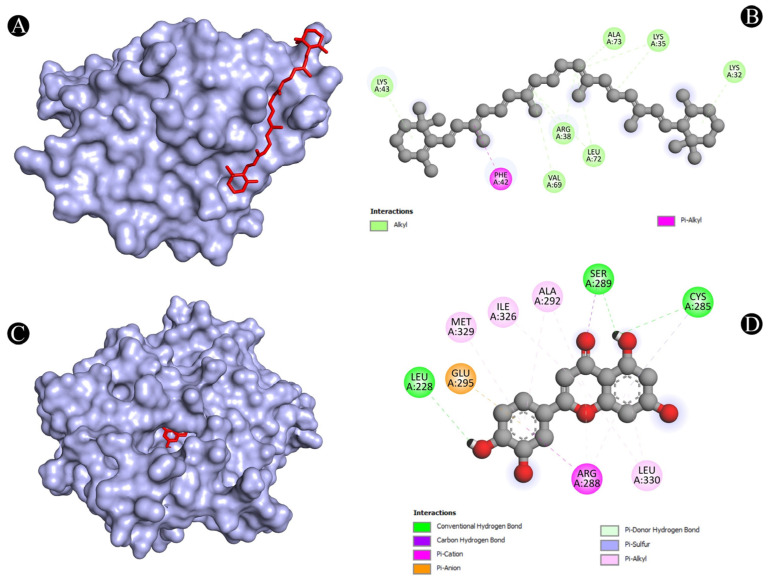
(**A**,**B**) Visualization of docking analysis of IL-2 and β-carotene, and (**C**,**D**) visualization of docking analysis of PPARG and luteolin.

**Figure 10:**
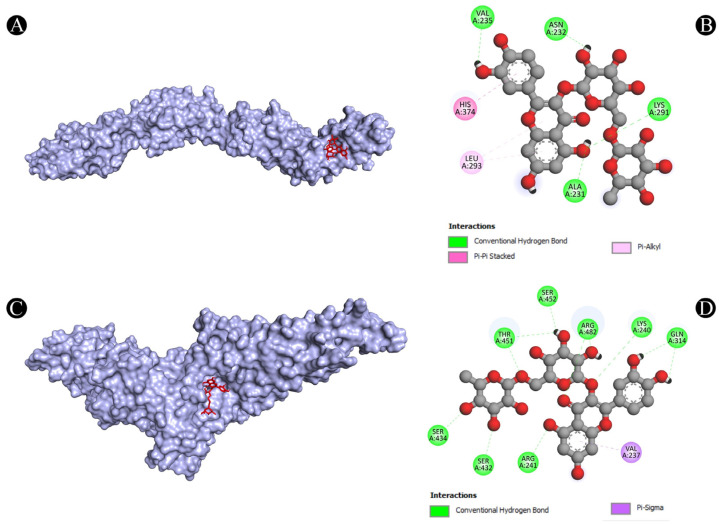
(**A**,**B**) Visualization of docking analysis of PTPRC and rutin, and (**C**,**D**) visualization of docking analysis of STAT1 and rutin.

**Figure 11:**
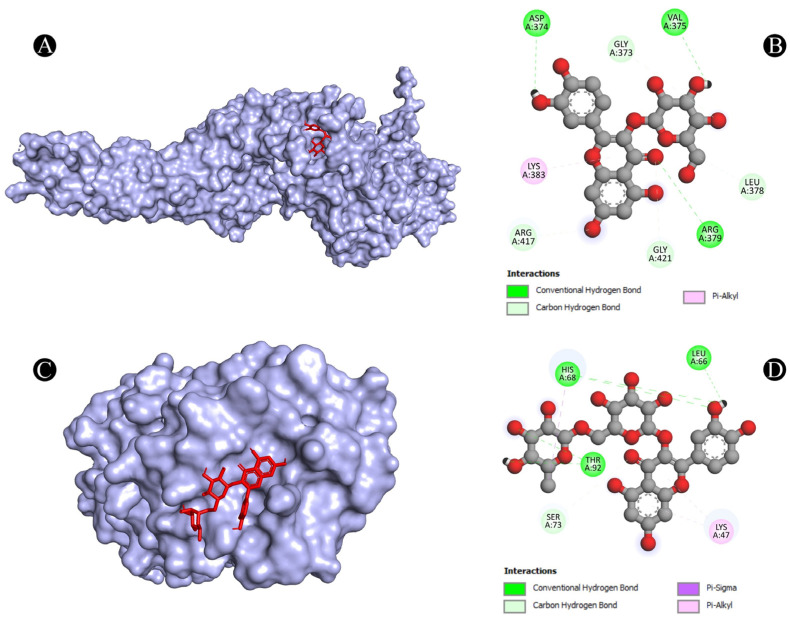
(**A**,**B**) Visualization of docking analysis of STAT3 and isoquercetin, and (**C**,**D**) visualization of docking analysis of TLR4 and rutin.

**Figure 12:**
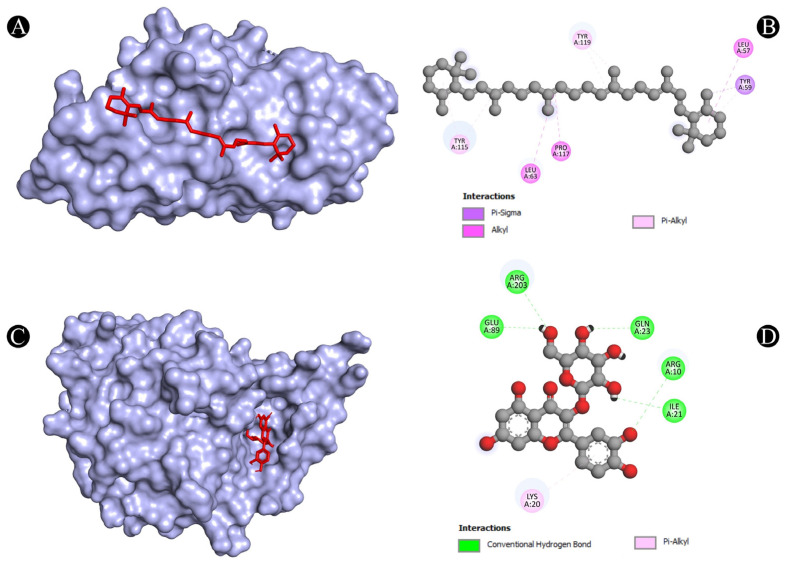
(**A**,**B**) Visualization of docking analysis of TNF and β-carotene, and (**C**,**D**) visualization of docking analysis of TP53 and isoquercetin.

As one of the most popular computational methods for finding potential leads against predefined targets, virtual screening using molecular docking is an effective strategy. The application of this method resulted in the identification of compounds with high binding affinities and specific interactions with the target proteins. Molecular docking analysis was conducted in order to understand how phytochemical constituents of Ajwa dates interact with identified protein targets of humans that play a key role in the development of candidiasis. In a docking analysis, several compounds were found to significantly bind to the target proteins of humans (Figure 7). The highest binding affinity was found between ALB–rutin (−9.7 kJ/mol), STAT1–rutin (−9.2 kJ/mol), STAT3–isoquercetin (−8.7 kJ/mol), IL2–β-carotene (−8.5 kJ/mol), CASP1–β-carotene (−8.2 kJ/mol), TP53–isoquercetin (−8.8 kJ/mol), PPARG–luteolin (−8.3 kJ/mol), TNF– βcarotene (−7.7 kJ/mol), TLR4–rutin (−7.4 kJ/mol) and PTPRC–rutin (−7.0 kJ/mol). The results of rutin showed good binding energy towards ALB (−9.7 kcal/mol), showing four conventional hydrogen bonds (PRO110, 2*ASP108 and GLU425), two carbon–hydrogen bonds (ARG145 and GLU425), two pi-pi t-shaped bond (2*HIS146), and three pi-alkyl bonds (2*ARG145 and ARG114), towards STAT1 (−9.2 kcal/mol) with 14 conventional hydrogen bonds (LYS240, 2*ARG241, SER432, SER434, 3*THR451, 3*ARG482, 2*GLN314, SER452 and VAL237) and one pi-sigma bond (VAL237), towards PTPRC (−7.0 kcal/mol) with four conventional hydrogen bond (LYS291, VAL235, ALA231 and ASN232), one pip-pi stacked bond (HIS374) and two pi-alkyl bonds (2*LEU293), β-carotene showed good binding affinity towards IL2 (−8.5 kcal/mol) with ten alkyl bonds (LYS32, 2*LYS35, 2*ARG38, LYS43, VAL69, 2*LEU72, ALA73 and PHE42) and one pi-alkyl bond (PHE42), towards CASP1 (−8.2 kcal/mol) with one pi-sigma bond (TRP145), five alkyl bonds (2*ILE155, PRO277, VAL279 and ILE280) and five pi-alkyl bonds (4*TRP145 and TYR153), towards TNF (−7.7 kcal/mol) with one pi-sigma bond (TYR59), three alkyl bonds (LEU57, LEU63 and PRO117) and five pi-alkyl bonds (TYR59, 2*TYR115 and 2*TYR119), iso-quercetin showed good binding affinity towards TP53 (−8.8 kcal/mol) with five conventional hydrogen bonds (ARG10, ARG203, GLU89, GLN23 and ILE21), and one pi-alkyl bond (LYS20), towards STAT3 (−8.7 kcal/mol) with three conventional hydrogen bonds (ARG379, VAL375 and ASP374), four carbon hydrogen bonds (GLY373, ARG417, GLY421 and LEU378) and one pi-alkyl bond (LYS383), and luteolin showed good binding affinity towards PPARG (−8.3 kcal/mol) with three conventional hydrogen bonds (LEU228, CYS285 and SER289), one carbon hydrogen bond (SER289), one pi-cation bond (ARG288), one pi-anion bond (GLU295), one pi-donor hydrogen bond (ARG288), one pi-sulfur bond (CYS285) and eight pi-alkyl bonds (2*ARG288, 2*ALA292, ILE326, 2*LEU330 and MET329). As is shown in Figure 8, Figure 9, Figure 10, Figure 11 and Figure 12 and Table 4, the compounds were found to interact with target proteins of humans against candidiasis by occupying different sites.

### 3.5. MD Simulation Analysis

**Figure 13:**
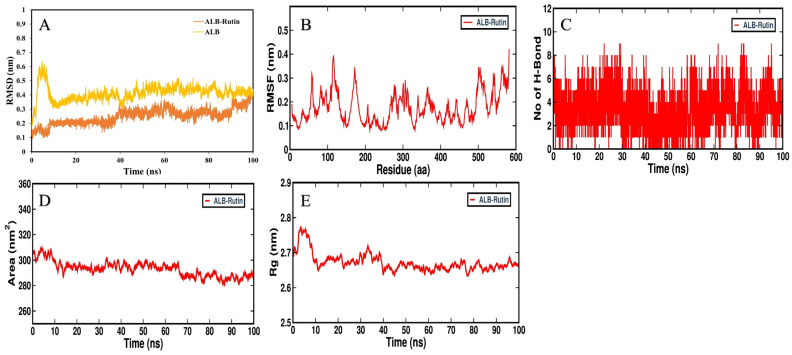
Molecular dynamics of ALB and its binding with rutin. (**A**) RMSD plot of ALB before and after rutin binding, (**B**) RMSF plot of ALB–rutin complex, (**C**) time evolution of intermolecular H-bonds formed within 0.35 nm in the ALB–rutin complex, (**D**) Rg distribution of ALB–rutin complex and (**E**) SASA plot analysis of ALB–rutin complex.

**Figure 14:**
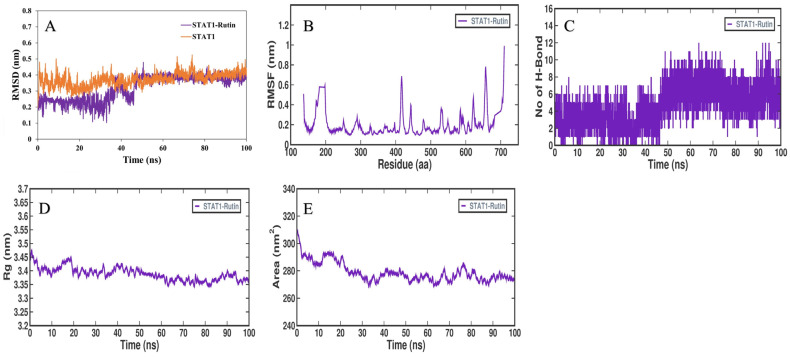
Molecular dynamics of STAT1 and its binding with rutin. (**A**). RMSD plot of STAT1 before and after rutin binding, (**B**). RMSF plot of STAT1–rutin complex, (**C**). Time evolution of intermolecular H-bonds formed within 0.35 nm between STAT1–rutin complex, (**D**). The Rg distribution of STAT1–rutin complex, (**E**). SASA plot analysis of STAT1–rutin complex.

To clarify the protein–ligand stability and protein structural flexibility between the docked complex of rutin–ALB and rutin–STAT1, further MD simulation using GROMACS software version 2019.4 was performed at 100 ns. Proteins and protein–ligand complexes can be examined using RMSD analysis to determine structure deviations. During the simulation, structural deviations of ALB, STAT1, ALB–rutin and STAT1–rutin complexes were investigated in the solvent environment to determine their stability and movement. As a result of the simulation, RMSD values of the backbone of ALB and the docked complex with rutin showed a stable pattern (Figure 13A). ALB and the ALB–rutin complex showed an average RMSD of 0.41 nm and 0.25 nm, respectively. As a result of the initial adjustments, random fluctuations in the RMSD pattern were seen in ALB systems between 0 and 10 ns. RMSD values of the backbone of STAT1 and the docked complex with rutin showed a stable pattern (Figure 14A). STAT1 and the STAT1–rutin complex showed an average RMSD of 0.41 nm and 0.37 nm, respectively. Throughout the simulation, the distribution of the RMSD pattern did not show any significant shifts, which suggested that ALB was stable amid a strong ligand binding strength during the simulation. The RMSF is an indicator of the flexibility of each residue in a protein. There was an average fluctuation of 0.17 nm in ALB–rutin (Figure 13B) and of 0.19 nm in the STAT1–rutin (Figure 14B) complex during the simulation. Following rutin binding, the fluctuations appeared stable and were minimized. Based on the graph, it appears that ALB and STAT1 with rutin interact with remarkable constancy. Protein structures depend on H-bonds for stability and integrity. In order to assess the structural integrity and stability of protein–ligand complexes, it is helpful to examine the time evolution of the formation and breakdown of H-bonds in the duration of the simulation. The intermolecular hydrogen bonds formed within the docked ALB–rutin and STAT1–rutin complex promote the stability of the protein and its ligand. ALB–rutin docked complex was maintained by six H-bonds (Figure 13C) and STAT1–rutin was maintained by seven H-bonds (Figure 14C). The simulation was therefore carried out in order to examine their time evolution during the simulation process. SASA refers to the surface area of a protein molecule that is accessible to its neighboring solvent. During simulations, SASA analysis is widely used to examine protein folding or unfolding and structural stability. Based on the simulation, there were no major peaks in SASA values, indicating that rutin binding affected ALB and STAT1 folding behavior. For ALB–rutin (Figure 13D) and STAT1–rutin (Figure 14D), the average SASA value was 292.98 and 278.90 nm^2^. SASA values showed that ALB and STAT1 was remained stable in the presence of rutin. Molecular stability can also be calculated from the compactness of protein molecules. The compactness measure in MD simulations is called Rg. The compactness of a protein structure is a useful parameter that can be used to examine the tertiary structure. Rg values were used to assess the compactness of ALB and STAT1 after daidzein binding. The ALB–rutin (Figure 13E) and STAT1–rutin (Figure 14E) complexes had an average Rg value of 2.67 and 3.38 nm. The Rg plot indicates that the protein–ligand complex remained compact throughout the simulation without significant changes.

### 3.6. MMPBSA Binding Free Energy

From the MD trajectory analysis, the binding free energy of rutin was estimated. There is an appreciable binding affinity of rutin for ALB and STAT1, i.e., −62.263 ± 45.849 and −111.180 ± 17.610 kJ/mol, respectively. In the study of MMPBSA, the results confirmed the observation that rutin was able to bind with ALB and STAT1 with an appreciable binding affinity.

## 4. Discussion

Candidiasis, a fungal infection caused in humans by various species of the *Candida* genus, represents a growing global health concern [1,2,3]. While conventional anti-fungal therapies have been instrumental in managing candidiasis, emerging challenges such as anti-fungal resistance, limited treatment options, and adverse effects have underscored the urgent need for innovative and effective approaches [17,18,20]. In this context, the exploration of natural compounds as potential candidates for candidiasis treatment has received significant attention. The appeal of natural compounds lies in their rich chemical diversity, often harboring complex bioactive molecules with distinctive mechanisms of action [51,54,55]. These molecules have the potential to target fungal pathogens via various routes, including disrupting cell membranes, inhibiting essential metabolic pathways, and modulating host immune responses [56,57,58]. Their multifaceted nature offers a complementary approach to combatting candidiasis in humans, potentially circumventing resistance mechanisms and broadening the spectrum of activity across various *Candida* species. Furthermore, the safety profile of natural compounds is often favorable, particularly when compared to that of synthetic anti-fungal agents that may carry risks of adverse effects. This attribute is particularly advantageous for vulnerable populations, such as pregnant women, children, and individuals with compromised immune systems, who require treatments with minimal side effects [59,60]. 

Incorporating traditional knowledge and indigenous practices into modern research adds a cultural dimension to the exploration of natural compounds [61]. Centuries of traditional medicine have often harnessed the healing potential of these compounds for various ailments, including fungal infections [62]. This accumulated information provides a foundation for identifying potential candidates and refining strategies for candidiasis treatment [63,64]. The present study employs an integrative network pharmacology approach to unravel the multi-target pharmacological mechanism underlying the antimicrobial activity of Ajwa dates against candidiasis in humans. This approach utilized different computational analyses to shed light on the complex interactions between bioactive compounds of Ajwa dates and potential target proteins in humans against candidiasis. The findings provide insights into the potential of Ajwa dates and their phytochemical constituents as novel antimicrobials and offer a blueprint for future investigations into natural products for combating fungal infections in humans.

We note that we have not found reports of interactions of the Ajwa date compounds with the target proteins we have identified. Nevertheless, some studies have shown that Ajwa dates and their seeds have anti-fungal properties against *C. albicans*. A study by Hussain et al. (2019) [65] compared the phenolic composition and antimicrobial activity of different Emirati date pits, including those of Ajwa. They found that Ajwa date pits had high levels of total phenolic acids and flavonoids, which may contribute to their antimicrobial properties. They also found that Ajwa dates pits inhibited *C. albicans* with an inhibition zone diameter of 15 mm and a minimum inhibitory concentration of 7.5 mg/mL. Selim et al. (2021) [66] isolated gallic acid from Ajwa dates pits and evaluated its activity against *C. albicans*, finding that gallic acid inhibited *C. albicans* with an inhibition zone diameter of 18 mm and a minimum inhibitory concentration of 5 mg/mL. In 2021, the authors of [28] reported the inhibition of the growth of *C. albicans* and *A. niger* with MIC values of less than 50 μg/mL by the polyphenol extract of Ajwa dates. In 2022, the authors of [67] also reported the anti-fungal activity of Ajwa date seed extract against *C. albicans*, where the former inhibited the latter’s growth and biofilm formation. 

In this study, we applied an integrative network pharmacology approach to identify the potential targets of the phytochemical constituents of Ajwa dates and their relevance to candidiasis in humans. This comprehensive approach allows for the prediction of multiple human targets for candidiasis, such as STAT3, IL-2, PTPRC, STAT1, CASP1, ALB, TP53, TLR4, TNF and PPARG. STAT3 is a transcription factor that regulates the differentiation and function of Th17 cells, which are essential for the defense against *Candida* infections on mucosal surfaces. However, excessive STAT3 activation can also impair anti-fungal immunity by suppressing other immune cells in humans. IL-2 is a cytokine that stimulates the proliferation and activation of T cells and Tregs [68,69]. IL-2 enhances the anti-fungal activity of T cells and macrophages, but also increases the number and function of Tregs, which can inhibit anti-fungal immunity in humans [70]. PTPRC is a protein tyrosine phosphatase that dephosphorylates various signaling molecules on immune cells. PTPRC can either enhance or inhibit anti-fungal immunity depending on the cell type and context. For example, PTPRC enhances the activation and proliferation of T cells, but inhibits the production of pro-inflammatory cytokines by macrophages [71,72]. STAT1 is another transcription factor that mediates the signaling of various cytokines, such as IFN-gamma and IL-27. STAT1 is essential for anti-fungal immunity, inducing genes involved in inflammation, cell-mediated immunity, and antimicrobial activity. STAT1 activates macrophages and neutrophils to kill *Candida* and promotes the differentiation and function of Th1 cells [73]. CASP1 is a protease that cleaves pro-inflammatory cytokines such as IL-1beta and IL-18 into their active forms. CASP1 plays a key role in innate immunity to candidiasis by inducing pyroptosis, which is a form of inflammatory cell death that releases cytokines and alarmins. CASP1 also enhances adaptive immunity to candidiasis by promoting the differentiation and function of Th17 cells [74]. ALB is the most abundant protein in blood plasma, where it transports various substances and has immunomodulatory effects. ALB can affect immunity to candidiasis in humans via different ways. On one hand, ALB can enhance anti-fungal immunity by increasing the production of pro-inflammatory cytokines by macrophages [75]. TP53 is a transcription factor that regulates various cellular processes, such as the cell cycle, apoptosis, DNA repair and senescence. TP53 is best known for its role in tumor suppression, but it is also involved in the immune response to candidiasis, modulating genes related to inflammation, cell-mediated immunity, and antimicrobial activity. TP53 can enhances anti-fungal immunity by increasing the production of pro-inflammatory cytokines by macrophages and promoting the activation and proliferation of T cells [76]. TLR4 is a transmembrane protein that recognizes LPS and other microbial or endogenous ligands. TLR4 is important for innate immunity to candidiasis, inducing the production of pro-inflammatory cytokines by macrophages and dendritic cells. TLR4 also enhances adaptive immunity to candidiasis by promoting the differentiation and function of Th17 cells [77]. TNF is a cytokine that mediates inflammation and immunity. TNF is essential for anti-fungal immunity, activating macrophages and neutrophils to kill *Candida* and stimulating the recruitment and extravasation of immune cells to the site of infection. TNF also regulates the differentiation and function of Th1 and Th17 cells. PPARG is a nuclear receptor that regulates various metabolic processes and has anti-inflammatory effects [78]. PPARG can modulate immunity to candidiasis in different ways. On one hand, PPARG can impair anti-fungal immunity by suppressing the activation and effector function of macrophages and T cells. On the other hand, PPARG can also protect against excessive inflammation and tissue damage caused by an uncontrolled immune response to *Candida* infection in humans [79].

Based on the GO analysis, possible targets of phytochemical constituents of Ajwa dates against candidiasis in humans are involved in multiple important GO processes, such as the regulation of the response to a cytokine stimulus, regulation of inflammatory responses, regulation of the cellular response to an external stimulus, etc, whereas according to the KEGG pathway analysis, potential targets of phytochemical constituents of Ajwa dates against candidiasis are significantly enriched in several important pathways, such as Th17 cell differentiation, the Toll-like receptor signaling pathway, the C-type lectin receptor signaling pathway and necroptosis. Th17 cell differentiation is a process by which naive CD4^+^ T cells develop into Th17 cells, a subset of T helper cells that produce interleukin-17 (IL-17) and other pro-inflammatory cytokines. Th17 cells play an important role in the host defense against fungal infections, such as candidiasis, by stimulating the production of other cytokines, such as TNF-α, IL-6, and IL-1β, and enhancing the recruitment and activation of neutrophils, which are essential for killing *Candida* cells [80]. Th17 cell differentiation is triggered by various cytokines and transcription factors that regulate the expression of RORγt (RAR-related orphan receptor gamma), the master regulator of Th17 cell fate. Some of the key cytokines involved in this pathway are IL-6, IL-21, IL-23, IL-1β and TGF-β [81].

The Toll-like receptor (TLR) signaling pathway is a mechanism by which the innate immune system recognizes and responds to microbial pathogens, such as *Candida* species. TLRs are a family of transmembrane proteins that can recognize specific molecular patterns derived from microbes, such as lipopolysaccharide (LPS), lipoteichoic acid (LTA), peptidoglycan (PGN), flagellin, zymosan and nucleic acids. These patterns are called microbe-associated molecular patterns (MAMPs) or pathogen-associated molecular patterns (PAMPs) [82]. The binding of ligands to TLRs activates specific intracellular signaling cascades that initiate host defense reactions. Depending on the type of TLR and ligand, different signaling adaptors are recruited to the TIR domain of TLRs. The most common adaptor is myeloid differentiation primary response gene 88 (MyD88), which is used by all TLRs except TLR3. MyD88-dependent signaling leads to the activation of nuclear factor kappa B (NF-κB) and mitogen-activated protein kinases (MAPKs), which are transcription factors that regulate the expression of pro-inflammatory cytokines [83]. Another adaptor is TIR-domain-containing adapter-inducing interferon-β (TRIF), which is used by TLR3 and TLR4. TRIF-dependent signaling leads to the activation of interferon regulatory factor 3 (IRF3) and IRF7, which are transcription factors that regulate the expression of type I interferons [83].

The C-type lectin receptor (CLR) signaling pathway is another mechanism by which the innate immune system recognizes and responds to fungal pathogens, such as *Candida* species. CLRs are a group of PRRs that have a carbohydrate recognition domain that can bind to specific sugar moieties on the surface of microbes. Some of the CLRs involved in anti-fungal immunity are Dectin-1, Dectin-2, Mincle, DC-SIGN and MBL [84]. The binding of ligands to CLRs activates various intracellular signaling pathways that modulate the immune responses. For example, Dectin-1 signaling leads to the activation of NF-κB and MAPKs through the Syk–CARD9–Bcl10–Malt1 complex [85]. Dectin-1 signaling also induces the production of reactive oxygen species (ROS) through the NADPH oxidase complex [86]. Moreover, Dectin-1 signaling cooperates with TLR2 signaling to enhance the production of pro-inflammatory cytokines and type I interferons [86]. Necroptosis is a type of programmed cell death that is mediated by the receptor-interacting protein kinases RIPK1 and RIPK3, and the mixed lineage kinase domain-like protein MLKL. Necroptosis is characterized by cell swelling, plasma membrane rupture and the release of intracellular contents, which can trigger inflammation and immune responses. Necroptosis can be induced by various stimuli, such as tumor necrosis factor (TNF), Toll-like receptors (TLRs), interferons, and intracellular RNA and DNA sensors [87]. *Candida* species can activate different TLRs and induce necroptosis in macrophages and dendritic cells [88]. Necroptosis can enhance anti-fungal immunity by releasing damage-associated molecular patterns (DAMPs) that stimulate the adaptive immune system and promote the clearance of *Candida* cells [89]. Therefore, it is found from the present study that Ajwa dates’ phytochemical constituents can fight against candidiasis through different pathways and mechanisms in humans (Table 5). 

Moreover, the results of molecular docking analysis confirmed that phytochemical constituents of Ajwa dates can bind stably to the active pockets of identified target proteins. Therefore, these compounds could be considered for use as a potential treatment for candidiasis in humans via modulating proteins such as, STAT3, IL-2, PTPRC, STAT1, CASP1, ALB, TP53, TLR4, TNF and PPARG. The further binding stability of the protein–ligand complex confirmed via molecular dynamics analysis revealed that these complexes display a stable conformation in solvation in water at a temperature of 300 K and at an atmosphere pressure of 1. This is in line with what was shown in the docking analysis. During the MD simulation, hydrogen bonds were found to be formed in protein–ligand complexes, which indicates that the interaction has a high level of affinity. As a result of this consideration of the importance of network pharmacology, the present study examines the phytochemical constituents of Ajwa dates, as well as their potential targets, pathways, and effects, as they relate to the treatment of candidiasis, thereby providing a theoretical foundation for further research in the future. Considering that network pharmacology has certain limitations, it is only through data mining that it is possible to identify the basic pharmacological mechanisms that are responsible for the treatment of candidiasis. The analysis of the bioactive properties of molecules in the context of network pharmacology is currently supported by a variety of databases. It is inevitable that there will be discrepancies between the information sources in a database since there are many different sources of information and experimental data. It is important to note that although we have presented some interesting results, the potential of Ajwa dates to serve as a preventive measure against candidiasis and other fungal diseases in humans still needs to be evaluated in further research and clinical trials.

## 5. Conclusions

In the present study, a multifaceted approach was used to investigate the anti-candidiasis potential of phytochemicals derived from Ajwa dates (*Phoenix dactylifera* L.) in humans through the integration of network pharmacology, molecular docking and dynamics simulations. The findings collectively shed light on the promising attributes of Ajwa date phytoconstituents as potential anti-fungal agents against *Candida* infections in humans. Through network pharmacology analysis, a complex interaction between Ajwa dates’ phytochemicals and *Candida*-associated molecular targets was identified. This approach allowed us to identify key compounds that may exert significant modulatory effects on fungal pathways. Subsequent molecular docking studies reinforced these interactions, highlighting the binding affinities of specific phytochemicals towards selected *Candida* protein targets of humans. The dynamics simulations provided deeper insights into the stability and dynamics of the ligand–target complexes, offering a comprehensive understanding of the binding mechanisms and intermolecular interactions governing the efficacy of the identified compounds. Overall, the present study underscores the potential of Ajwa dates’ phytochemicals as a source of anti-fungal agents for candidiasis treatment. By integrating computational techniques, the present study accelerated the process of identifying and characterizing potential therapeutic candidates. While findings of the present study build a promising foundation, further experimental validation and in vitro studies are essential to confirm the efficacy and safety of these compounds.

## Figures and Tables

**Table 1:** List of selected phytochemical constituents of Ajwa dates with their basic information and structure.

Sr. No.	Name	PubChem ID	MF	MW	Canonical SMILES	Structure
1	Apigenin	5280443	C_15_H_10_O_5_	270.24	C1=CC(=CC=C1C2=CC(=O)C3=C(C=C(C=C3O2)O)O)O	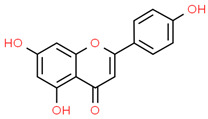
2	Caffeic acid	689043	C_9_H_8_O_4_	180.16	C1=CC(=C(C=C1C=CC(=O)O)O)O	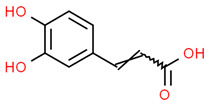
3	Catechin	9064	C_15_H_14_O_6_	290.27	C1C(C(OC2=CC(=CC(=C21)O)O)C3=CC(=C(C=C3)O)O)O	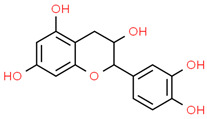
4	Chlorogenic acid	1794427	C_16_H_18_O_9_	354.31	C1C(C(C(CC1(C(=O)O)O)OC(=O)C=CC2=CC(=C(C=C2)O)O)O)O	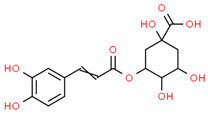
5	Digalacturonic acid	439694	C_12_H_18_O_13_	370.26	C1(C(C(OC(C1O)OC2C(C(C(OC2C(=O)O)O)O)O)C(=O)O)O)O	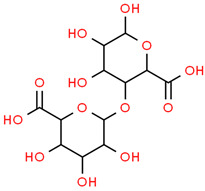
6	Ferullic acid	445858	C_10_H_10_O_4_	194.18	COC1=C(C=CC(=C1)C=CC(=O)O)O	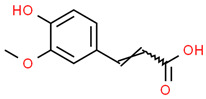
7	Gallic acid	370	C_7_H_6_O_5_	170.12	C1=C(C=C(C(=C1O)O)O)C(=O)O	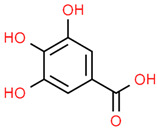
8	Iso-quercetin	10813969	C_21_H_20_O_12_	464.4	C1=CC(=C(C=C1C2=C(C(=O)C3=C(C=C(C=C3O2)O)O)OC4C(C(C(C(O4)CO)O)O)O)O)O	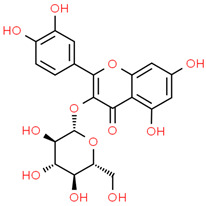
9	Luteolin	5280445	C_15_H_10_O_6_	286.24	C1=CC(=C(C=C1C2=CC(=O)C3=C(C=C(C=C3O2)O)O)O)O	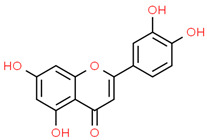
10	Myricetin	5281672	C_15_H_10_O_8_	318.23	C1=C(C=C(C(=C1O)O)O)C2=C(C(=O)C3=C(C=C(C=C3O2)O)O)O	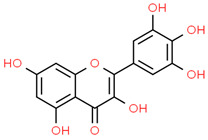
11	p-coumaric acid	637542	C_9_H_8_O_3_	164.16	C1=CC(=CC=C1C=CC(=O)O)O	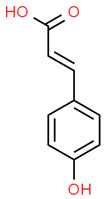
12	Protocatechuic acid	72	C_7_H_6_O_4_	154.12	C1=CC(=C(C=C1C(=O)O)O)O	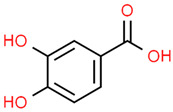
13	Quercetin	5280343	C_15_H_10_O_7_	302.23	C1=CC(=C(C=C1C2=C(C(=O)C3=C(C=C(C=C3O2)O)O)O)O)O	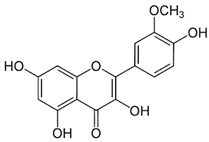
14	Resorcinol	5054	C_6_H_6_O_2_	110.11	C1=CC(=CC(=C1)O)O	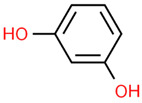
15	Rutin	5280805	C_27_H_30_O_16_	610.5	CC1C(C(C(C(O1)OCC2C(C(C(C(O2)OC3=C(OC4=CC(=CC(=C4C3=O)O)O)C5=CC(=C(C=C5)O)O)O)O)O)O)O)O	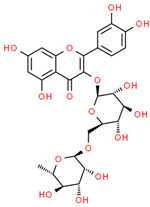
16	β-Carotene	5280489	C_40_H_56_	536.9	CC1=C(C(CCC1)(C)C)C=CC(=CC=CC(=CC=CC=C(C)C=CC=C(C)C=CC2=C(CCCC2(C)C)C)C)C	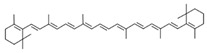
17	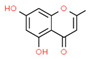	222284	C_29_H_50_O	414.7	CCC(CCC(C)C1CCC2C1(CCC3C2CC=C4C3(CCC(C4)O)C)C)C(C)C	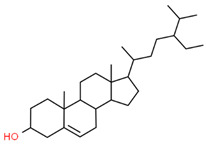

**Table 2:** Topological parameters of the targeted proteins.

Sr. No.	Genes	Degree	Betweenness	Closeness
1	TNF	65	988.208	0.15648286
2	ALB	64	1225.0367	0.15695067
3	STAT3	54	470.05515	0.15418503
4	EGFR	53	668.1908	0.15441176
5	VEGFA	50	370.19366	0.15328467
6	TP53	50	373.23392	0.15328467
7	TLR4	47	222.14207	0.15239477
8	PTPRC	45	248.6846	0.15086207
9	IL2	45	268.14774	0.1517341
10	STAT1	42	167.48207	0.15086207
11	HRAS	41	266.0002	0.15129682
12	ICAM1	38	276.51984	0.1502146
13	HSP90AA1	37	441.82166	0.15064563
14	PPARG	35	175.21269	0.14957266
15	SYK	33	68.06617	0.14767933
16	CCL5	33	62.759113	0.14767933
17	ERBB2	33	128.60736	0.14872521
18	ESR1	32	262.75586	0.14957266
19	MAPK14	32	76.563156	0.14872521
20	MPO	32	104.47263	0.1480959
21	CAT	30	590.64325	0.14872521
22	SELL	29	68.562836	0.14623955
23	LCK	28	69.942894	0.1474719
24	CASP1	27	38.12457	0.1474719
25	NR3C1	26	135.64508	0.1474719
26	ITGB2	25	38.64269	0.1446281
27	SELE	24	31.730501	0.14623955
28	SELP	24	28.364697	0.14563107
29	BTK	24	38.74685	0.14502762
30	TLR9	23	26.082947	0.14522822
31	IKBKB	23	16.278957	0.14644352
32	HDAC1	23	75.05072	0.14644352
33	PLG	23	93.70508	0.14664805
34	HSP90AB1	23	156.0722	0.1474719
35	REN	22	101.38719	0.14583333
36	ZAP70	22	22.437016	0.14482759
37	ABCB1	21	110.38468	0.14644352
38	RAC2	21	30.51389	0.14383562
39	ELANE	20	15.914155	0.14344262
40	LGALS3	20	203.58853	0.1446281
41	CXCR1	20	11.40124	0.14403293
42	ITGAL	20	17.016296	0.14363885
43	CYP3A4	19	90.02866	0.14603616
44	F2	19	33.436974	0.14403293
45	F3	18	12.540235	0.14403293
46	JAK3	16	12.340351	0.14363885
47	HSPA8	16	51.118656	0.1446281
48	PTPN6	15	3.2728631	0.1420839
49	CTSG	15	10.223254	0.14227642
50	LCN2	15	11.1281185	0.14383562
51	SOD2	14	26.54373	0.1446281
52	SHH	14	19.630491	0.14403293
53	ADAM17	13	3.4592445	0.14324693
54	PTPN22	13	3.366081	0.1418919
55	FGFR1	13	4.2498856	0.14383562
56	CYP1A2	13	239.40514	0.14246947
57	HDAC2	13	5.7672634	0.14383562
58	CD209	13	4.428888	0.14266305
59	FGF1	13	7.1389694	0.14423077
60	IKBKG	13	2.9129455	0.14285715
61	HPRT1	13	41.86046	0.14403293
62	FGFR2	13	62.313843	0.14285715
63	IDO1	12	114.35072	0.14363885
64	WAS	12	9.96579	0.13962767
65	CYP2C9	12	28.406013	0.14131898
66	EPHA2	12	110.74736	0.1420839
67	CHEK1	11	3.897872	0.14344262
68	COMT	11	44.608868	0.13636364
69	ATP12A	11	20.04787	0.14266305
70	ITK	11	2.3593173	0.13962767
71	DHFR	10	143.46443	0.14324693
72	CYP2C19	10	51.41783	0.14056225
73	ICAM2	10	1.6470731	0.13981359
74	CYP17A1	9	132.31921	0.136897
75	RNASE3	8	0.6450318	0.14075068
76	MAPK10	7	1.7650008	0.13833992
77	UGT2B7	7	2.0575106	0.13944224
78	LTF	7	0.71694976	0.14018692
79	CYP51A1	6	265.89664	0.13530928
80	RORC	6	0	0.13981359
81	TGM2	6	8.930667	0.14112903
82	ADA	6	15.780002	0.14112903
83	TNK2	6	3.9851854	0.1392573
84	IL6ST	5	0	0.13779527
85	LAP3	5	98.4974	0.13707572
86	MPI	5	223.96904	0.13027295
87	PNP	4	83.11256	0.136897
88	ACADM	4	6.846439	0.13358779
89	CFB	4	0.9110306	0.13981359
90	DYRK1A	4	1.15	0.13636364
91	APCS	3	0	0.13907285
92	GALK1	3	19.146414	0.12962963
93	TPH1	3	5.359037	0.13092269
94	SQLE	3	14.4429655	0.12382075
95	RAN	3	0	0.13307984
96	FDFT1	2	0	0.12041284
97	CRAT	2	0	0.13059701
98	TREH	1	0	0.13043478
99	CHIT1	1	0	0.13636364
100	ARSA	1	0	0.00952381
101	ECE1	1	0	0.12727273
102	PSAP	1	0	0.00952381
103	GNPDA1	1	0	0.1160221
104	CA6	0	0	0.009433962
105	PNPO	0	0	0.009433962
106	AKR1A1	0	0	0.009433962

**Table 3:** Topological parameters of phytochemical constituents of Ajwa dates.

Sr. No.	Compounds	Degree	Betweenness	Closeness
1	Myrecitin	52	551.76263	0.49593496
2	Quercitin	51	523.5583	0.4919355
3	Luteolin	52	652.4729	0.49593496
4	Rutin	57	1270.8419	0.5169492
5	Isoquercitin	54	855.9138	0.5041322
6	Chlorogenic acid	58	1955.7657	0.52136755
7	Catechin	47	422.1807	0.4765625
8	Digalacturonic acid	56	1521.1764	0.5126051
9	Apigenin	46	393.6386	0.4728682
10	Ferrulic acid	51	1359.5	0.4919355
11	Caffeic acid	47	1025.8472	0.4765625
12	Gallic acid	44	779.46545	0.46564886
13	Protocatechuic acid	33	176.04279	0.42957747
14	Beta-sitosterol	43	1956.6543	0.46212122
15	p-coumaric acid	38	838.4169	0.4452555
16	Beta-carotene	35	881.8214	0.43571427
17	Resorcinol	20	932.9409	0.3935484

**Table 4:** Interaction analysis of receptor ligand.

Sr. No.	Protein	Receptor-Ligand	Interaction Type	Distance
1	ALB	N:UNK1:H-A:PRO110:O	Conventional Hydrogen Bond	2.41382
		N:UNK1:H-A:ASP108:O	Conventional Hydrogen Bond	1.97207
		N:UNK1:H-A:GLU425:OE2	Conventional Hydrogen Bond	2.20851
		N:UNK1:H-A:ASP108:O	Conventional Hydrogen Bond	2.60039
		A:ARG145:CD-N:UNK1:O	Carbon Hydrogen Bond	3.09589
		N:UNK1:C-A:GLU425:OE2	Carbon Hydrogen Bond	3.09465
		A:HIS146-N:UNK1	Pi-Pi T-Shaped	5.79161
		A:HIS146-N:UNK1	Pi-Pi T-Shaped	5.91519
		N:UNK1-A:ARG145	Pi-Alkyl	5.23581
		N:UNK1-A:ARG114	Pi-Alkyl	5.19604
		N:UNK1-A:ARG145	Pi-Alkyl	5.49631
2	IL2	A:LYS32-N:UNK1	Alkyl	4.38514
		A:LYS35-N:UNK1	Alkyl	4.59242
		A:LYS35-N:UNK1	Alkyl	3.78304
		A:ARG38-N:UNK1	Alkyl	4.70114
		A:ARG38-N:UNK1	Alkyl	4.64384
		A:LYS43-N:UNK1	Alkyl	5.14946
		A:VAL69-N:UNK1	Alkyl	5.48053
		A:LEU72-N:UNK1	Alkyl	4.86731
		A:ALA73-N:UNK1	Alkyl	4.04445
		N:UNK1-A:LEU72	Alkyl	4.75444
		A:PHE42-N:UNK1	Pi-Alkyl	4.28876
3	PPARG	N:UNK1:H-A:LEU228:O	Conventional Hydrogen Bond	3.05297
		N:UNK1:H-A:CYS285:O	Conventional Hydrogen Bond	2.84417
		N:UNK1:H-A:SER289:OG	Conventional Hydrogen Bond	2.46012
		A:SER289:CA-N:UNK1:O	Carbon Hydrogen Bond	3.46788
		A:ARG288:NH2-N:UNK1	Pi-Cation	3.74404
		A:GLU295:OE1-N:UNK1	Pi-Anion	4.02798
		A:ARG288:NE-N:UNK1	Pi-Donor Hydrogen Bond	3.91198
		A:CYS285:SG-N:UNK1	Pi-Sulfur	5.52645
		N:UNK1-A:ARG288	Pi-Alkyl	4.10762
		N:UNK1-A:ALA292	Pi-Alkyl	4.4279
		N:UNK1-A:ILE326	Pi-Alkyl	5.37149
		N:UNK1-A:LEU330	Pi-Alkyl	5.03093
		N:UNK1-A:ARG288	Pi-Alkyl	3.59494
		N:UNK1-A:LEU330	Pi-Alkyl	4.98253
		N:UNK1-A:ALA292	Pi-Alkyl	5.35981
		N:UNK1-A:MET329	Pi-Alkyl	4.6949
4	PTPRC	A:LYS291:HZ1-N:UNK1:O	Conventional Hydrogen Bond	2.49711
		N:UNK1:H-A:VAL235:O	Conventional Hydrogen Bond	2.70571
		N:UNK1:H-A:ALA231:O	Conventional Hydrogen Bond	2.54842
		N:UNK1:H-A:ASN232:O	Conventional Hydrogen Bond	2.57383
		A:HIS374-N:UNK1	Pi-Pi Stacked	4.36158
		N:UNK1-A:LEU293	Pi-Alkyl	5.26513
		N:UNK1-A:LEU293	Pi-Alkyl	5.26961
5	STAT1	A:LYS240:HZ3-N:UNK1:O	Conventional Hydrogen Bond	2.11392
		A:ARG241:HH11-N:UNK1:O	Conventional Hydrogen Bond	2.78186
		A:ARG241:HH21-N:UNK1:O	Conventional Hydrogen Bond	1.92015
		A:SER432:HG-N:UNK1:O	Conventional Hydrogen Bond	2.58764
		A:SER434:HG-N:UNK1:O	Conventional Hydrogen Bond	2.76816
		A:THR451:HG1-N:UNK1:O	Conventional Hydrogen Bond	3.06789
		A:ARG482:HH11-N:UNK1:O	Conventional Hydrogen Bond	2.16428
		A:ARG482:HH11-N:UNK1:O	Conventional Hydrogen Bond	3.0504
		A:ARG482:HH21-N:UNK1:O	Conventional Hydrogen Bond	2.62021
		N:UNK1:H-A:GLN314:O	Conventional Hydrogen Bond	2.12922
		N:UNK1:H-A:GLN314:O	Conventional Hydrogen Bond	2.11387
		N:UNK1:H-A:THR451:O	Conventional Hydrogen Bond	2.07658
		N:UNK1:H-A:THR451:OG1	Conventional Hydrogen Bond	2.24927
		N:UNK1:H-A:SER452:O	Conventional Hydrogen Bond	2.74446
		A:VAL237:CG2-N:UNK1	Pi-Sigma	3.65444
6	STAT3	A:ARG379:NH1-N:UNK1:O	Conventional Hydrogen Bond	3.33961
		N:UNK1:H-A:VAL375:O	Conventional Hydrogen Bond	2.64317
		N:UNK1:H-A:ASP374:OD1	Conventional Hydrogen Bond	2.35408
		A:GLY373:CA-N:UNK1:O	Carbon Hydrogen Bond	3.45037
		A:ARG417:CA-N:UNK1:O	Carbon Hydrogen Bond	3.42956
		A:GLY421:CA-N:UNK1:O	Carbon Hydrogen Bond	3.39518
		N:UNK1:C-A:LEU378:O	Carbon Hydrogen Bond	3.29566
		N:UNK1-A:LYS383	Pi-Alkyl	5.00243
7	TLR4	A:HIS68:ND1-N:UNK1:O	Conventional Hydrogen Bond	3.03529
		A:SER73:CA-N:UNK1:O	Carbon Hydrogen Bond	2.94848
		A:THR92:OG1-N:UNK1:O	Conventional Hydrogen Bond	3.05155
		N:UNK1-A:LYS47	Pi-Alkyl	5.33037
		N:UNK1-A:LYS47	Pi-Alkyl	5.15142
		N:UNK1:C-A:HIS68	Pi-Sigma	3.88961
		N:UNK1:H-A:HIS68:O	Conventional Hydrogen Bond	2.20639
		N:UNK1:H-A:LEU66:O	Conventional Hydrogen Bond	2.34857
		N:UNK1:H-A:THR92:OG1	Conventional Hydrogen Bond	2.39563
8	TNF	N:UNK1:C-A:TYR59	Pi-Sigma	3.73712
		A:LEU57-N:UNK1	Alkyl	5.38917
		A:LEU63-N:UNK1	Alkyl	5.26338
		A:PRO117-N:UNK1	Alkyl	4.92982
		A:TYR59-N:UNK1	Pi-Alkyl	4.35802
		A:TYR115-N:UNK1	Pi-Alkyl	4.90592
		A:TYR115-N:UNK1	Pi-Alkyl	4.49189
		A:TYR119-N:UNK1	Pi-Alkyl	4.33167
		A:TYR119-N:UNK1:C	Pi-Alkyl	4.13411
9	TP53	A:ARG10:NH1-N:UNK1:O	Conventional Hydrogen Bond	3.15168
		A:ARG203:HH2-N:UNK1:O	Conventional Hydrogen Bond	2.79577
		N:UNK1:H-A:GLU89:OE2	Conventional Hydrogen Bond	2.30967
		N:UNK1:H-A:GLN23:O	Conventional Hydrogen Bond	2.28776
		N:UNK1:H-A:ILE21:O	Conventional Hydrogen Bond	2.7139
		N:UNK1-A:LYS20	Pi-Alkyl	5.35537
10	CASP1	N:UNK1:C-A:TRP145	Pi-Sigma	3.79529
		A:ILE155-N:UNK1	Alkyl	5.20732
		A:ILE155-N:UNK1	Alkyl	4.76342
		A:PRO277-N:UNK1	Alkyl	4.22891
		A:VAL279-N:UNK1	Alkyl	4.21566
		A:ILE280-N:UNK1	Alkyl	5.42589
		A:TRP145-N:UNK1	Pi-Alkyl	5.22269
		A:TRP145-N:UNK1	Pi-Alkyl	4.00488
		A:TRP145-N:UNK1	Pi-Alkyl	4.62996
		A:TRP145-N:UNK1	Pi-Alkyl	5.03064
		A:TYR153-N:UNK1	Pi-Alkyl	5.27671

**Table 5:** Biochemical pathways targeted by Ajwa date extract and its potential against fungal infection and virulence factors.

Biochemical Pathway	Effect on Fungus or Virulence Factor	Explanation	References
STAT3 (signal transducer and activator of transcription 3)	Suppresses inflammation and tissue damage caused by fungal infection	STAT3 is a transcription factor that can modulate the immune response and prevent excessive inflammation and tissue damage. STAT3 can also inhibit the growth and invasion of *C. albicans* by regulating the expression of anti-fungal genes and enhancing the phagocytosis of fungal cells. Ajwa date extract may stimulate the release of STAT3 and enhance its anti-fungal activity.	[90,91]
IL-2 (interleukin-2)	Promotes T cell activation and proliferation against fungal infection	IL-2 is a cytokine that can stimulate the activation and proliferation of T cells, which are immune cells that can recognize and kill infected cells. IL-2 can also enhance the production of other cytokines that have anti-fungal effects, such as IFN-gamma and TNF-alpha. Ajwa date extract may stimulate the release of IL-2 and increase its anti-fungal function.	[69,70]
PTPRC (protein tyrosine phosphatase receptor type C)	Regulates T cell receptor signaling and immune response against fungal infection	PTPRC, also known as CD45, is a protein that can regulate the signaling of T cell receptor (TCR), which is a molecule that recognizes antigens presented by infected cells. PTPRC can modulate the activation and differentiation of T cells and their anti-fungal effector functions. Ajwa date extract may stimulate the release of PTPRC and improve its anti-fungal function.	[71,72]
STAT1 (signal transducer and activator of transcription 1)	Activates anti-fungal genes and enhances the phagocytosis of fungal cells	STAT1 is a transcription factor that can activate the expression of genes that are involved in anti-fungal responses, such as IFN-gamma, NOS2 and CXCL10. STAT1 can also enhance the phagocytosis of fungal cells by macrophages, which are immune cells that can engulf and destroy foreign particles. Ajwa date extract may stimulate the release of STAT1 and increase its anti-fungal function.	[90,92]
CASP1 (caspase-1)	Induces the pyroptosis (inflammatory cell death) of infected cells and prevents fungal dissemination	CASP1 is a protein that can trigger pyroptosis, which is a process of inflammatory cell death, in the response to fungal infection. Pyroptosis can help eliminate infected cells and prevent the spread of fungal pathogens. Pyroptosis can also release cytokines, such as IL-1beta and IL-18, that have anti-fungal effects. Ajwa date extract may stimulate the release of CASP1 and increase its pyroptotic function.	[74]
ALB (albumin)	Binds to fungal toxins and neutralizes their effects	ALB is a protein that can bind to various substances in the blood, including fungal toxins such as gliotoxin and fumagillin. ALB can neutralize the effects of these toxins on immune cells and tissues. Ajwa date extract may stimulate the release of ALB and enhance its anti-toxin activity.	[75]
TP53 (tumor protein p53)	Induces the apoptosis (cell death) of infected cells and prevents fungal dissemination	TP53 is a protein that can trigger apoptosis, which is a process of programmed cell death, in response to DNA damage or stress. Apoptosis can help eliminate infected cells and prevent the spread of fungal pathogens. Ajwa date extract may stimulate the release of TP53 and increase its apoptotic function.	[93]
TLR4 (Toll-like receptor 4)	Recognizes fungal components and activates the inflammatory response against fungal infection	TLR4 is a protein that can recognize fungal components such as lipopolysaccharide and beta-glucan. TLR4 can activate the inflammatory response against fungal infection by inducing the expression of cytokines such as TNF-alpha, IL-1beta, IL-6, IL-12 and IL-23. Ajwa date extract may stimulate the release of TLR4 and increase its anti-fungal function.	[77]
TNF (tumor necrosis factor)	Induces inflammation and cell death against fungal infection	TNF is a cytokine that can induce inflammation and cell death against fungal infection by activating the expression of genes such as NOS2, CXCL10 and ICAM1. TNF can also enhance the phagocytosis of fungal cells by macrophages and neutrophils. Ajwa date extract may stimulate the release of TNF and increase its anti-fungal function.	[78]
PPARG (peroxisome proliferator-activated receptor gamma)	Inhibits fungal growth and biofilm formation	PPARG is a protein that can inhibit the growth and biofilm formation of *C. albicans* by regulating the expression of genes such as EFG1, NRG1 and HWP1. PPARG can also modulate the immune response and inflammation against fungal infection by influencing the production of cytokines such as IL-10, IL-17 and TGF-beta. Ajwa date extract may stimulate the release of PPARG and increase its anti-fungal function.	[79]

## Data Availability

All data generated and analyzed during the course of this study are included in the article.
